# Prevalence of Iron Deficiency and Anemia among Young Children with Acute Diarrhea in Bhaktapur, Nepal

**DOI:** 10.3390/healthcare3030593

**Published:** 2015-07-21

**Authors:** Ram K. Chandyo, Manjeswori Ulak, Ramesh K. Adhikari, Halvor Sommerfelt, Tor A. Strand

**Affiliations:** 1 Centre for International Health, University of Bergen, Overlege Danielsens Hus, 5 et. Årstadveien 21, N-5009 Bergen, Norway; E-Mails: halvor.sommerfelt@uib.no (H.S.); tor.strand@uib.no (T.A.S.); 2 Community Medicine Department, Kathmandu Medical College, Kathmandu University, Kathmandu P.O. Box 21266, Nepal; 3 Department of Child Health, Institute of Medicine, Kathmandu P.O. Box 1524, Nepal; E-Mail: manjeswori@gmail.com; 4 Department of Pediatrics, Kathmandu Medical College, Kathmandu University, Kathmandu P.O. Box 21266, Nepal; E-Mail: ramesh497@gmail.com; 5 Medical Microbiology, Innlandet Hospital Trust, Lillehammer N-2629, Norway

**Keywords:** iron deficiency, anemia, diarrhea, children, Nepal

## Abstract

Iron deficiency anemia is still common in children under five years of age and may impair their growth and cognitive development. Diarrhea is the second most common reason for seeking medical care for young children in Nepal. However, neither screening programs nor effective preventive measures for anemia and iron deficiencies are in place among children with diarrhea in many developing countries. The aims of this study were to determine the prevalence of anemia and iron deficiency and explore their associations with clinical, socioeconomic, and anthropometric parameters in Nepalese children. This was a cross-sectional study based on 1232 children, six to 35 months old, with acute diarrhea participating in a zinc supplementation trial. The mean (SD) hemoglobin was 11.2 g/dL (1.2). Anemia was found in 493 children (40%); this estimate increased to 641 (52%) when we adjusted for the altitude of the study area (hemoglobin <11.3 g/dL). One in every three children had depleted iron stores and 198 (16%) of the children had both depleted iron stores and anemia, indicating iron deficiency anemia. The prevalence of anemia among children presenting with acute diarrhea was high but the degree of severity was mainly mild or moderate. Iron deficiency explained less than half of the total anemia, indicating other nutritional deficiencies inducing anemia might be common in this population.

## 1. Introduction

Iron deficiency (ID) is still a common nutritional deficiency in developing countries responsible for more than 50% of total anemia cases in children under 5 [1]. Iron deficiency anemia (IDA), which is a severe form of ID, is associated with impairment of motor development and cognitive performance, as well as fatigue, sleep disturbance, irritability, and poor memory and school performance [2,3,4]. Clinical or subclinical inflammation may also cause anemia, which is common among children in resource-poor settings [5,6]. An optimal balance of iron in our body is essential and both excess and deficiency may be harmful [7]. Thus, more population-based information on iron status data is warranted for the development of an effective nutritional program to combat ID [7].

Children, particularly young ones, are more susceptible to anemia and ID because of high iron requirements during growth, low intake of iron from complementary foods, and frequent episodes of infection [8]. Breast milk contains relatively low levels of iron but it is readily absorbable and sufficient for infants up to six months of age [9,10,11]. However, a longer duration of breastfeeding may increase the risk for ID [12,13]. Extra iron is required from six months of age onward, either from complementary foods or as a supplement [14]. A traditional Nepali diet and Nepali complementary foods are usually monotonous cereal (rice) based and contain very few nutrients from animal sources [15]. In the last national survey of 2011 among children under 12 months, only about 13% were reported as having consumed iron-rich foods (meat, fish, poultry, or eggs) in the previous 24 h [16]. Grains, in particular rice, which provides most of the total calories in the diet, contain low bioavailable iron and high phytate, which interferes with iron absorption [15,17]. The absorption of bioavailable iron of foods depends not only on its actual content but also on vitamin C, the amount that is bound to heme iron, and also on the status of body iron stores.

Due to its simplicity of use in field settings and low cost, anemia detected by only hemoglobin (Hb) measurement has been widely used as a proxy for detection of ID and IDA [14]. Although ID is a major cause of nutritional anemia in developing countries, deficiencies of other nutrients like vitamin A, C, B9, B12, vitamin D, and zinc as well as toxicity of lead may also cause anemia [18,19,20,21] and Hb alone is a poor predictor for detection of iron status [22,23]. Moreover, the detection of anemia and ID may be obscured due to clinical or sub-clinical infections and concomitant multiple micronutrient deficiencies, which are common in developing countries [24].

In Nepal, there is not a regular screening program for anemia and ID in children and population-based data on anemia, including ferritin concentration in children, are still scarce. Detection of anemia is usually done when a child is attending a health center for other illnesses. Diarrhea is, after fever, the main presenting symptom in young Nepali children visiting health centers [16]. This paper focuses on the prevalence of anemia and iron deficiency and explores their associations with socioeconomic and anthropometric parameters in children of 6–35 months old with acute diarrhea, who were taking part in a zinc intervention trial in Bhaktapur, Nepal during June 1998–September 2000. The main outcomes of this trial have been published elsewhere [25,26].

## 2. Experimental Section

### 2.1. Study Area and Recruitment Procedure

The study participants were residents of Bhaktapur municipality, which is a semi-urban city in Kathmandu valley. Bhaktapur is situated approximately 1400 m above sea level. There are approximately 80,000 inhabitants in Bhaktapur municipality and most people have agriculture as their main occupation. The municipality is one of the most densely populated areas in Nepal with 11,058 people per sq km [27]. The houses are mostly attached to each other and made of brick and mud. Most of the traditional residents of Bhaktapur (80%) belong to the Newar ethnic group and are non-vegetarian but consumption of meat products is infrequent and usually only when there are local festivals or celebrations. Local vegetables including leafy green vegetables are abundant during seasons, and are usually grown on their own land. The main sources of income are daily wage earning, selling of agricultural products, self-employment in small scale businesses, and service in government or private sector. The study had ethical clearance from the institutional review board, Institute of Medicine, Kathmandu, Nepal and the Human Research Ethical Committee of the Medical Faculty at the University of Bergen.

This was a cross-sectional study where we collected blood samples between June 1998 and September 2000 from 1792 children. Children aged six to 35 months with acute diarrhea were recruited through weekly surveillance or spontaneous visits to a field hospital. This was the only pediatric hospital in the community and during the study period we had regular weekly household surveillance visits so we believe that we picked up most of the diarrheal illness in the community*.* Anemic children got extra attention from a study physician and, if required, were treated with iron and anti-helmenthic drugs according to the national guidelines.

### 2.2. Data Collection

We weighed the children with minimal clothes at enrollment using a scale with 100 g sensitivity (Salter, SECA, Hamburg, Germany) and measured length or height using a locally made wooden board, which read to the nearest 0.1 cm. We collected baseline blood from the cubital vein into heparinized polypropylene tubes (Sarstedt, Nümbrecht, Germany) irrespective of feeding status. The Hb concentration was analyzed immediately after blood collection with HemoCue (Ångelholm City, Sweden) according to the manufacturer’s guidelines. The heparinized blood was centrifuged for 10 minutes and plasma was separated. The specimens were transported to Norway on dry ice and stored at −70 °C for 3–4 years before analysis. The plasma ferritin and C-reactive protein (CRP) were analyzed by a turbidimetric immunoassay (Tina-Quant, Roche, Mannheim , Germany) on a Hitachi 917 (Tokyo, Japan) at the Laboratory of Clinical Biochemistry, Haukeland University Hospital in Bergen, Norway. We analyzed Hb from 1419 children as Hb measurement was not possible in the first ~300 enrollments because of logistical problems. Some of the samples did not contain enough volume to obtain reliable estimates of ferritin and CRP concentration. The analyses and statistical tests were thus based on the 1232 children for whom results for CRP, ferritin, and Hb are available.

### 2.3. Definitions

According to the WHO, Hb concentration <11 g/dL in younger children is defined as anemia and severe anemia when Hb was lower than 7 g/dL [14]. The Centers for Disease Control (CDC) have developed altitude-specific adjustment of Hb to get a proper diagnosis of anemia for people residing at a higher altitude like in Bhaktapur (1400 m) [28]. We therefore also reported data based on an altitude-adjusted cutoff (Hb <11.3 g/dL). Depleted iron stores was defined as plasma ferritin <12 μg/L [14]. Plasma ferritin concentrations are highly vulnerable to change during infection or inflammation [29]. Thus, we also presented our analyses that include ferritin in children with CRP <10 mg/L. IDA was considered when anemia was associated with depleted iron stores and possible anemia due to other causes was considered when it was not associated with depleted iron stores. Definition of diarrheal illnesses and categorizations of dehydration were done according to the WHO/IMCI guidelines [30].

### 2.4. Statistical Analysis

The Hb concentrations were symmetrically distributed. The distribution of plasma ferritin was skewed towards higher values. The descriptive statistics included means, medians, interquartile ranges, and 95% confidence intervals as appropriate. Student *t*-test or Chi-square tests were used to determine the difference between Hb and ferritin with clinical or other baseline features. Anemia and depleted iron stores were used as dependent variables in crude and in multiple logistic regression analyses to determine its relation with other variables. As we enrolled children with acute diarrhea, possible confounding factors like the baseline number of diarrheal stools and dehydration as well as CRP concentration were adjusted in the multiple regression analyses*.* Based on their associations and relevance, a total of five variables (age of child, stunting, numbers of stool >10 times, family ownership of land, and birth order) were selected for the final model in the logistic regression analyses. We considered *p*-values of less than 0.05 to represent statistical significance. The z-scores of weight for age, length, or height for age were calculated using the WHO growth chart [31]. The statistical analyses were undertaken using Stata^®^, version 10.1 (STATA Corp, Houston, TX, USA).

## 3. Results and Discussion

### 3.1. Baseline Features and Association with Hb and Ferritin Concentration

A total of 1792 children were enrolled in the study and analysis of Hb and ferritin was based on 1232 children. The mean age was 15.4 months (SD 7.7) and 40% (*n* = 494) were infants (Table 1). Overall, 85% children were still breastfeeding at the time of enrolment but almost all (99%) infants below one year of age were breastfeeding. About half of the total children (*n* = 634) were from joint families. The age of the mothers of the enrolled children ranged from 17 to 45 years (mean ± SD = 25 ± 4.6) and 16% (*n* = 201) were 20 or younger. Nearly half of the children’s mothers (*n* = 599) had no formal education and another 20% (*n* = 241) had just primary level or informal education. Most of the women (*n* = 665) were not engaged in any formal job but one in every three spent more than 5 h per day working away from home, particularly in agricultural work. Although most of the children (95%) below one year of age were introduced to semi-solid foods, particularly rice, only one-third of parents reported giving animal or formula milk to their children.

**Table 1:** Baseline characteristics among children evaluating for anemia and iron deficiency in Bhaktapur, Nepal ^1^.

Characteristics	*n* = 560	*n* = 1232
Mean age, months (SD)	15.7 (7.8)	15.4 (7.7)
Male child	308 (55)	684 (55)
First-born child	220 (39)	471 (38)
Mean number of loose stools 24 h prior to enrolment (SD)	**9.3 (4.3)**	**8.7 (3.9)**
Some dehydration ^2^	**90 (16)**	**118 (10)**
Stunted (<−2Z length for age)	**187 (33)**	**341 (28)**
Wasted (<−2Z weight for length)	127 (23)	271 (22)
Underweight (<−2Z weight for age)	279 (50)	565 (46)
Breast feeding infants ^3^	**225 (96)**	**491 (99)**
Infants introduced to animal or formula milk ^3^	**93 (40)**	**158 (28)**
Infants introduced to solid or semisolid foods ^3^	211 (91)	524 (95)
Family having drinking water from a tap	535 (96)	1175 (95)
Family having toilet	544 (97)	1175 (95)
Cemented household	126 (24)	296 (24)

^1^: Values are n (%), unless otherwise mentioned. Baseline characteristics were compared between children available for blood sampling (*n* = 1232) and children not available for blood sampling due to not enough blood (*n* = 560). Figures in bold indicate *p*-value < 0.05; ^2^: Defined according to World Health Organization/IMCI guidelines; ^3^: Among infants <12 months of age, *n* = 494.

Underweight (low weight for age) was the most common form of malnutrition affecting almost every second child (46%), followed by stunting (low height or length for age) and wasting (low weight for height or length). The mean duration of diarrhea before enrollment was 2.2 (SD 1.1) days and the mean number of stools was 8.7 (SD 3.9) times during the preceding 24 h prior to the enrollment. Approximately one in every 10 children had blood in the stool or some signs of dehydration. The mean total number of stools 24 h prior to the enrolment was 9.0 among children who were anemic and 8.6 among non-anemic (*p* = 0.05). However, there were no apparent associations between Hb with proportion of children with blood in stool, dehydration status, and post-enrollment diarrheal durations.

The mean plasma ferritin concentration among children with blood in the stool (21.1 μg/L *vs.* 17.4 μg/L, *p* = 0.0001) or axillary temperature >99 °F (20.6 μg/L *vs.* 17.4 μg/L, *p* = 0.0002) was significantly higher than in children without blood in the stool or temperature ≤99 °F, respectively. Children with a higher CRP (>10 mg/L) also had significantly higher plasma ferritin concentration (21.6 μg/L *vs.* 16.1 μg/L, *p* = 0.0001) but there were not significant differences in Hb concentration (11.1 g/dL *vs.* 11.2 g/dL, *p* = 0.08).

### 3.2. Prevalence of Anemia and Depletion of Iron Stores

The mean values and 95% CI of Hb and ferritin concentrations are presented in Table 2. The overall prevalence of anemia (Hb < 11 g/dL) was 40% (*n* = 493) and after adjusting for the altitude of the study area (1400m), it became 52% (*n* = 641). Among the infants below one year of age, the anemia prevalence was 54% (*n* = 267), which is substantially higher than the 31% (*n* = 226) prevalence in older children (*p* = 0.0001). The overall prevalence of depleted iron stores (plasma ferritin <12 μg/L) was 33% (*n* = 407), but when we restricted the analyses to those with CRP concentration of <10 mg/L (*n* = 867), it increased to 39% (*n* = 340). The mean ferritin concentration was not significantly different between infants and older children. Out of a total of 1232 children, 43% (*n* = 530) had normal Hb and ferritin concentrations, 17% (*n* = 209) were iron deficient but not anemic, probably indicating a transient stage of iron deficient erythropoiesis, and 24% (*n* = 295) were anemic but not iron deficient, possibly due to other causes of anemia. A total of 198 children (16%) were found to be both anemic and ID, which is according to our definition considered as IDA. Severe anemia was found in only one child, indicating that most of the anemia in this population was of a mild and moderate type.

**Table 2:** Mean hemoglobin and plasma levels of ferritin and prevalence of anemia and iron deficiency among children aged 6–35 months with acute diarrhea in Bhaktapur, Nepal.

Biochemical Markers	N (1232)	Cutoff Values	Results
Mean (SD) hemoglobin level, g/dL			11.2 (1.2)
Anemia	493	11 g/dL	40%
Anemia adjusted to altitude ^1^	641	11.3 g/dL	52%
Median (IQR) plasma ferritin, μg/L			16.0 (10, 25)
Depleted iron stores	407	12 μg/L	33%
Depleted iron stores among children with CRP <10 mg/L ^2^	340	12 μg/L	39%
Iron deficiency anemia ^3^	198		16%
Anemia without iron deficiency	295		24%

^1^: 0.3 g/dL of Hb value is added for the altitude (1400 m) of Bhaktapur; ^2^: Among children with CRP <10 mg/L (*n* = 867); ^3^: Defined as anemia with the depleted iron stores (plasma ferritin <12 μg/L).

### 3.3. Correlation and Regression Analyses

Spearman’s rank order correlation analysis showed a positive association between Hb and plasma ferritin (r = 0.18, *p* = 0.0001) and age (r = 0.36, *p* = 0.0001). Most of the infants (84%) were enrolled during spring or summer and post-monsoon months as compared with winter months (October–January), but the prevalence of anemia or depleted iron stores was not significantly associated with seasons. In the multiple logistic regression analysis, the odds of having anemia were lower in older children and in those who came from families that owned a piece of land (Table 3). Only the age of children older than 24 months significantly predicted depleted iron stores when it was used as a dependent variable in multiple regression analyses. Zinc deficiency (plasma zinc <10 μmol/dL) was found among 84% of children and the figure among anemic children was 85% (82% in non-anemic, *p* = 0.1); among children with depleted iron stores it was 87% (90% among non-depleted iron stores, *p* = 0.5).

**Table 3:** Multiple logistic regression for the association of age and nutritional status of children and socio-demographic characteristics with anemia and iron deficiency among children aged 6–35 months in Bhaktapur, Nepal.

			Anemia ^1^		Depleted Iron Stores ^1^		
Variables	*n* = 1232		Adjusted			Adjusted	
		OR^2^	95% CI	*p-*value	OR ^2^	95% CI	*p-*value
Age of children (Months)							
≤12	554 (45%)	1					
13–24	498 (40%)	0.31	(0.24, 0.40)	<0.0001	0.98	(0.77, 1.4)	0.9
>24	180 (15%)	0.21	(0.12, 0.35)	<0.0001	0.52	(0.32, 0.83)	0.006
Stunted (<2Z length for age)							
No	891 (72%)	1					
Yes	341 (28%)	0.97	(0.74, 1.3)	0.7	1.1	(0.83, 1.4)	0.4
Total number of stool >10 times in past 24 h							
No	946 (77%)	1					
Yes	286 (23%)	1.1	(0.83, 1.5)	0.5	0.91	(0.69, 1.2)	0.3
Family ownership of land							
No	360 (29%)	1					
Yes	872 (79%)	0.57	(0.44, 0.74)	<0.0001	0.95	(0.75, 1.2)	0.3
Birth order third or above							
No	880 (71%)	1					
Yes	352 (29%)	0.79	(0.60, 1.0)	0.08	0.78	(0.75, 1.2)	0.6
Seasons of enrolment ^3^							
Summer (monsoon)	520 (42%)	1			1		
Winter	189 (16%)	0.75	(0.53, 1.1)	0.1	0.78	(0.59, 1.0)	0.09
Spring	523 (42%)	0.88	(0.68, 1,1)	0.3	0.81	(0.63, 1.0)	0.08

^1^: Anemia was defined as hemoglobin <11.3 g/dL and depleted iron stores when plasma ferritin <12 μg/L; ^2^: Adjusted regression coefficient and *p* values obtained from logistic regression model adjusted for the variables included in this Table and breastfeeding, animal, or formula feeding and dehydration status. Age was used as a continuous variable; ^3^: Summer season is from June to September, winter from October to January, and spring from February to May.

Despite a substantial declining trend over the last decades, the current prevalence of anemia in Nepal is still high, affecting one in every two children under five years of age [16]. Our study, which included children agef 6–35 months with acute diarrhea, also showed that anemia is a common nutritional problem as 40% of the included children were anemic. A community-based study from the southern parts of Nepal reported an even higher prevalence of anemia of 78% (using a cutoff of Hb 11 g/dL) and IDA of 43% [32]. Unfortunately, there are not many published studies on anemia and ID in young Nepalese children and the available data are often based on a small number of participants or hospitalized children. Some available figures from adolescents in schools or refugee camps found that the prevalence of anemia ranged from 24% to 68% [33,34,35]. Many indicators of anemia and iron deficiency, particularly the ferritin concentration, are susceptible to change during an infection so caution is warranted when comparing our results to otherwise healthy children. Besides, our estimation of anemia and iron deficiency is based on children suffering from diarrhea and is not from a representative sample of infants, which may be one of the limiting factors when comparing our results with otherwise healthy infants. The volume of total body fluids and dehydration status influences the hemoglobin concentration [36], so our children with diarrhea, especially with signs of some dehydration, may have some degree of hemo concentration. However, we restricted the sample to children with normal CRP (<10 mg/L) when interpreting the value of ferritin and adjusted for dehydration status and stool frequency at baseline, which we believe to some extent took care of this possible source of bias. Diarrhea is still a major cause of morbidity and mortality in Nepal [37] and understanding anemia prevalence among these children will provide useful information for initiating preventive strategies. Thus, although our data are a bit old, we believe that the results can still contribute to a better understanding of the anemia problem among young children who are most vulnerable. Moreover, most of our children did not have any signs of dehydration and due to regular weekly household surveillance we believe that we picked up most of the diarrheal illness in the community, so our findings of anemia prevalence may resemble the figures from healthy children. The duration of exclusive breastfeeding may also influence the iron status of infants [12,38] but, unfortunately, we did not have information on exclusive breastfeeding in the first six months as we enrolled children six months or older.

Most anemia in developing countries is probably due to ID, though other nutritional or hematological abnormalities or parasitic infestation may also cause it [14]. The high burden of anemia among children is also due to inflammation (clinical or sub-clinical) [6], which is very common in resource-poor settings such as our study area [5]. Our study area is not endemic for malaria and hemoglobinopathies. Although hookworm is still common in some other parts of Nepal, it is not common in our study area. IDA is the end stage of a chronic process and reflects the tip of the “iceberg” problem; underlying sub-clinical ID is usually expected to be far more common in the population [39]. Indeed, it is estimated that the prevalence of ID is 2.5 times higher than the anemia prevalence [14]. However, we find this relation in our study as the prevalence of both depleted iron stores, which reflects ID, and anemia were almost identical. The transit stage of ID, also known as iron-deficient erythropoiesis, may be more common in this young population and further worsening of iron supply may lead to IDA. Or it could be due to the fact that ferritin concentrations are still falsely high even among those with a CRP concentration of less than 10 mg/L, the cutoff which we have used. Notably, for better estimating of plasma ferritin value during infection, the WHO suggests using a cutoff of 30 μg/L [14]. If we had used this cutoff, the prevalence of depleted iron stores would have been 84% (*n* = 1034), which is twice the prevalence of anemia and may be a true reflection of ID in this population. Another limitation of our study was that we did not measure zinc protophorphyrin, MCV, RBC, or plasma-soluble transferrin receptor, which can identify iron-deficient erythropoiesis as an early stage of ID and is not affected by infection [40,41].

The 16% prevalence of IDA, which explained less than 50% of the total anemia prevalence, indicates that other causes of anemia could also be prevalent in this population. Notwithstanding iron, several other nutrient deficiencies like folate, B12, zinc, vitamin A, vitamin D, and selenium have also been found to be associated with the development of anemia [42,43,44,45,46]. Similar anemia prevalence (but not accompanied by low ferritin concentration) was also observed among young Mexican infants [47]. Plasma zinc concentration was also associated with Hb concentration among pregnant Ethiopian women, indicating that zinc may be one of the nutrients causing anemia [48]. Indeed, zinc and vitamin B12 deficiencies were found to be common among these children [49] and women in our study area, affecting more than three-fourths of its population [50], but plasma zinc was not associated with the hemoglobin and ferritin concentrations in this population.

We used a portable Hemocue photometer for estimating Hb. This method has been widely used for the estimation of anemia prevalence in developing countries and is well validated for this purpose. The use of Hemocue on venous blood was recommended for the estimation of anemia in a hospital setting as estimation from capillary blood tends to give values that are 0.5 g/dL higher on average [51]. The time between breaking the seal of the microcuvette containers and hemoglobin assessment may also affect the readings. If the micrucuvette was used after the twelfth day of opening the seal of the container, then it overestimated the Hb concentration, which may be due to the hydroscopic nature of the cuvette in a humid environment [52]. The use of cuvettes in our study depended upon the rates of enrolment and unfortunately we did not record the day of opening the container seal and the time until the cuvette was used.

Traditionally, when the prevalence of anemia exceeds 40%, it is regarded as a serious public health problem, prompting interventions including fortification or universal iron supplementation without screening [53]. However, a recent finding on iron supplementation to healthy children did not favor this recommendation, particularly for children who were not iron deficient or in a population where infections such as malaria are common [54]. The revised recommendations now focus on targeted rather than universal iron supplementation for children [55]. Our results, which are based on blood analyses of more than 1000 children, also indicate that most of the observed anemia cases were mild or moderate and were not due to iron deficiency favoring a targeted rather than a universal approach of supplementation. Improvement in complementary feeding could also be one of the acceptable options to enhance the iron status in young children in a community [56]. Generally, in developing countries, foods used as complementary foods are not of high quality due to poverty, lack of knowledge, food taboos, and cultural practices [57].

Monotonous rice or other cereal-based complementary foods are predominantly used in Nepal and other developing countries and also have high phytate levels, leading to further inhibition of already compromised iron absorption [32,58]. Our finding of a positive association between ownership of land as a marker of socioeconomic status and anemia prevalence also indicated that children from these families get relatively diverse, iron-rich foods. Unfortunately, we did not collect systematic information on dietary habits including intake of iron-rich or -inhibiting foods from these children. Green leafy vegetables, fruits, yoghurt, and meat products are generally not given to young children in Nepal due to the belief that they are difficult to digest or because they may cause a cough and difficulty breathing [15]. Similarly, tea with milk is commonly consumed and given to infants from eight to 10 months of age [59]. Drinking tea may also interfere with the absorption of iron because of tannins, phytophenols, and calcium [60]. In our area, many give their infants eggs from around seven months of age [61]. Eggs are a good source of iron and are culturally acceptable and comparatively less expensive than other animal-based foods. Apart from this, introduction of anti-helmenthic drugs to children older than one year of age was also found to be effective in decreasing the anemia burden [62], but we are still not certain of its impact on infants younger than one year.

## 4. Conclusions

The proportion of anemia in children presenting with acute diarrhea was high but mostly mild or moderate. Iron deficiency anemia explained less than half of the total anemia burden, suggesting that other nutritional deficiencies inducing anemia might be common in this population.
